# Player Monitoring in Professional Soccer: Spikes in Acute:Chronic Workload Are Dissociated From Injury Occurrence

**DOI:** 10.3389/fspor.2020.00075

**Published:** 2020-07-08

**Authors:** Luis Suarez-Arrones, Borja De Alba, Mareike Röll, Ignacio Torreno, Sarah Strütt, Kathrin Freyler, Ramona Ritzmann

**Affiliations:** ^1^Section of Physical Education and Sports, Department of Sports and Computer Science, Universidad Pablo de Olavide, Seville, Spain; ^2^FC Basel 1893, Basel, Switzerland; ^3^FC Sevilla Performance Department, Seville, Spain; ^4^Department of Sport and Sport Science, University of Freiburg, Freiburg, Germany; ^5^Praxisklinik Rennbahn AG, Muttenz, Switzerland

**Keywords:** elite sport, distance, running, training, competition, match, contact injuries, non-contact injuries

## Abstract

This study aimed to determine whether spikes in acute:chronic workload ratio (ACWR) are associated with injury incidence, and to examine the differences in external load due to greater or lesser exposure to matches and the long-term effects of the load during a chronic seasonal period. Fifteen professional soccer players belonging to the squad of a European Champions League club were enrolled in this study. External training and match load were assessed from all athletes using a global positioning system (GPS). We calculated the uncoupled ACWR for 10 consecutive competitive microcycles. Injuries were identified and determined by the days of absence. The differences in external load were determined using a linear mixed-model approach. In addition to the null hypothesis testing, the effect size was calculated. Thirteen athletes who did not suffer an injury exceeded several times the critical threshold of an ACWR > 1.5. This is equivalent to 1 player exceeding the critical threshold for ACWR in total distance (TD), 2 players for ACWR at distance covered above moderate speed (MSD), 2 players for ACWR at distance covered above high speed (HSD), 2 players for ACWR at distance covered above very high speed (VHSD), and 2 players for ACWR in DC at sprint per week. One athlete experienced a non-contact muscle strain injury and another a contact -injury manifested as a concussion; both athletes document an ACWR <1.5 within the 4 weeks prior to the injury event. Players with lesser participation in official games covered lower TD (−19.6%, very-large ES), MSD (−24.8%, very-large ES), HSD (−25.1%, moderate ES), VHSD (−25.5%, moderate ES), and DC at sprint (−30.6%, moderate ES) over the course of the 10-weeks period in comparison with the players with greater participation in official games. The present study demonstrated that spikes in the ACWR were not related to a subsequent injury occurrence in professional soccer players. Differences in participation in official games caused significant imbalances in the chronic external loads between players in a squad, which should be minimized in training sessions in order to prevent substantial changes in workload for those who usually do not play.

## Introduction

Injuries are an important issue in professional football and they can negatively affect team performance, representing a significant cost to professional football clubs (Hagglund et al., 2013). Professional football players normally suffer two injuries *per season*; consequently a professional team can expect about 50 injuries *per season* (Ekstrand et al., 2011). Players availability is associated with team success; therefore, the protection of players' health by preventing injuries is claimed as essential by professional football clubs and international sports federations and is thus a crucial task for medical and trainer teams (Engebretsen et al., 2008; Hagglund et al., 2013). Injuries occur during a given workload in training or matches, and major changes in training load or an inappropriate workload are likely to increase the risk of injury.

Extensive research has been conducted to parameterize and predict individuals' risk of sustaining an injury using monitoring techniques (Fanchini et al., 2018). Monitoring training load helps in understanding the individual responses of athletes throughout the season and optimizing the training process (Impellizzeri et al., 2019). Measurements of internal training load quantify/assess the physiological and perceptual responses experienced during training sessions, and external training loads describe the running activity profiles of players during training sessions (Campos-Vazquez et al., 2014; Impellizzeri et al., 2019). The results of training are the consequence of both stimuli, and the monitoring of players' workload is crucial to understand the individual physiological responses and biological adaptations to training for optimal training load management (Campos-Vazquez et al., 2014; Coppalle et al., 2019). The use of global positioning system (GPS) technology to measure players' locomotor activity profiles during matches and training has become particularly prevalent in professional football (Suarez-Arrones et al., 2015; Torreno et al., 2016; Bowen et al., 2020), and GPS-based parameters (i.e., distance, speed) are established to objectively quantify external loads.

The use of the acute:chronic workload ratio (ACWR) to manage changes in load and how these changes are related to injury risk has received increasing scientific attention. A general model for the relationships between players' ACWRs and their risk of injury was generated using different units of load [i.e., balls bowled, distance run, accelerations, decelerations, session rating of perceived exertion (RPE)] and data from diverse team sports (Hulin et al., 2016; Bowen et al., 2017, 2020; Fanchini et al., 2018; Jaspers et al., 2018; Wang et al., 2020). The ACWR is calculated as the acute (i.e., recent) load divided by the chronic (i.e., long-term) load (Hulin et al., 2014; Impellizzeri et al., 2020) and was developed as a preventive model to predict injury. This tool indicates whether an athlete has sufficiently prepared for an upcoming acute load (normalized to their previous chronic loads) (Wang et al., 2020). The number of articles examining the relationships between training load and injuries has grown in recent years and now exceeds 100 publications (Impellizzeri et al., 2020). Previous studies in professional soccer and rugby players have shown that high chronic workloads with large spikes in acute workloads (high ACWR changes) are related to a high risk of injury (Gabbett, 2016; Hulin et al., 2016; Bowen et al., 2017, 2020). Authors still debate the optimal parameterization and critical thresholds of the ACWR in terms of which parameter combined with which zones of intensity are relevant to predicting injury. Recent studies have proposed external load parameters such as total distance (TD) (Hulin et al., 2016), high speed and sprint (Malone et al., 2017), decelerations (Bowen et al., 2020) and accelerations (Bowen et al., 2017) as strong indicators of overall and non-contact injury risk, identifying ACWR values >1.5 as dangerous acute spike workloads with a high injury risk (Gabbett, 2016). Contrary to the aforementioned findings, recent articles using internal load indicators suggest a poor interrelationship between the ACWR and injury incidence (Fanchini et al., 2018; Lolli et al., 2019). In professional soccer players, increased ACWRs were associated with poor predictive validity in identifying players who will suffer a future injury (Fanchini et al., 2018), and the relationships between match and training loads with hamstring injuries showed no associations between ACWRs and injury occurrence in professional football (Lolli et al., 2019).

Based on the scientific research there are disparities between studies that have described the relationship between ACWR and injury risk, and ACWR suffers from several serious limitations (Wang et al., 2020). A recent study showed that when the ACWR is used as an explanatory variable, results are always influenced by artifacts and artificial alterations; therefore ACWRs and its components should be dismissed (Impellizzeri et al., 2020). In addition, “injury-associated” and “injury-predicting” are different terms and should be used for different purposes (Bahr, 2016; Fanchini et al., 2018). Studies have showed that associations may not predict injuries (Pepe et al., 2004; Fanchini et al., 2018). In addition, important limitations exist in elite football for the assessment of GPS-derived external loads during the whole season (including individualization of relative running loads, external loads tracked with different systems, dealing with international breaks, and missing data) and it is stated that injury prevention can hardly be limited to the monitoring of a single ACWR number (Buchheit, 2017). Based on the training-injury prevention paradox and with reference to players' individual needs, an adequate load management with respect to physical demands in competitions, and subsequent choices based on an optimized training stimulus may be an appropriate option to keep players fit and healthy (Gabbett, 2016). The most important and specific training stimulus in team sports is the competition itself (Suarez-Arrones et al., 2015; Torreno et al., 2016; Al Haddad et al., 2018). Squads are composed of players with more and fewer minutes in competition; therefore, coaches are challenged to optimally manage the load for the football players who did not play, or those who played for only a few minutes, in order to balance the training load and avoid imbalances in chronic training loads and reductions in players' fitness (Martin-Garcia et al., 2018).

Despite the popularity of the ACWR, there is limited understanding of the imbalances in workload caused by differences in the external load in matches between players from the same team. Therefore, this manuscript aims to provide a contribution to the interface of spikes in ACWRs and likely subsequent injuries in light of training and competition in elite soccer. The purpose was to determine whether spikes in ACWR are associated with injury incidence, and to examine the differences in external load due to greater or lesser exposure to matches and the long-term effects of the load during a chronic seasonal period.

## Methods

### Experimental Design

An observational design was used to examine the external loads of soccer players during 10 full competitive microcycles (10 full training weeks with 10 official matches) using GPS technology during the 2018–2019 season. All matches and training sessions were performed on outdoor natural grass fields.

### Subjects

Fifteen professional outfield players [18.6 ± 0.8 years, 180.4 ± 3.6 cm, 76.2 ± 6.8 kg, 10.9 ± 1.2% body fat (Faulkner)] participated in this study. Players belonged to the reserve squad of a Spanish La Liga club that competed in the UEFA Champions League. The running sprint performance over 5-, 10-, and 20-m was 1.01 ± 0.03 s, 1.71 ± 0.09, s 2.96 ± 0.08 s; and the intermittent fitness performance (30–15_IFT_) (Buchheit, 2008) was 21.0 ± 0.6 km/h. Our data came from routine monitoring over the 2018–2019 season; therefore, institutional ethics committee authorization was not required (Winter and Maughan, 2009). The study conformed to the current national and international laws and regulations governing the use of human subjects (Declaration of Helsinki II), and the players provided informed consent before participating.

### Running Demands Analysis

The players were required to wear a GPS unit (EVO; GPSports Systems, Canberra, Australia) during all training sessions and official games, which was fitted to the upper back (i.e., between the shoulder blades) of each player using an adjustable neoprene harness. GPS data were recorded at a frequency of 10 Hz. The validity and reliability of the GPS system have been previously reported (Coutts and Duffield, 2010; Varley et al., 2012) and it has been used with soccer players during official games (Suarez-Arrones et al., 2015; Torreno et al., 2016; Al Haddad et al., 2018). Each player used the same device during the study period. The total distance (TD), distance covered (DC) above moderate speed (>14.4 km/h, MSD), DC at high speed (>18.0 km/h, HSD), DC at very high speed (>21.0 km/h, VHSD), and DC sprinting (>24 km/h) were quantified. The uncoupled ACWR (acute load is not in the chronic workload calculation) was calculated during 10 full competitive microcycles (for this, the 4 weeks prior to the first microcycle were also monitored to create the first ACWR value) using the different parameters analyzed. Chronic workload was calculated as the 4-weeka rolling average acute workload, and ACWR was calculated by dividing the acute workload by the chronic workload (load accumulated during the current week/previous four-week average). A value of >1.5 represented an acute spike workload with a high injury risk (danger zone) (Gabbett, 2016).

### Definition of Injury

Injury information was classified by a medical practitioner. A recordable injury was defined as one that caused an absence from future football participation; and injury incidence was reported in absolute numbers and as an injury incidence rate for number of injuries per 1,000 player hours during matches and on-field training sessions (Fuller et al., 2006). Injuries were classified as minimal (1–3 days of active participation missed), mild (4–7 days missed), moderate (1–4 weeks missed), or severe (4+ weeks missed) (Fuller et al., 2006). The mechanism by which a participant acquired an injury was classified as non-contact or contact in nature.

### Statistical Analyses

All statistical tests were performed using JMP Version 14.2.0 (SAS Institute Inc., Cary, NC, USA). If not otherwise indicated, data are presented as means ± standard deviation (SD), with statistical significance set at p ≤ 0.05. Descriptive statistics were calculated on each external load variable for players with lesser and greater participation in competitive matches. Shapiro–Wilk tests were used to verify normality. The differences in external load were determined using a linear mixed-model approach, with player and number of participations as random effects. In addition to the null hypothesis testing, the effect size (ES, 90% confidence interval [90%CI]) in the selected variables was calculated for possible differences between groups. Threshold values for assessing magnitudes of the ES (changes as a fraction or multiple of baseline SD) were <0.20, 0.20, 0.60, 1.2, and 2.0 and categorized as trivial, small, moderate, large, and very large, respectively (Hopkins et al., 2009). For further analysis, players were divided into two groups according to previous studies (Al Haddad et al., 2015; Suarez-Arrones et al., 2019) with lesser or greater participation in official games during the 10-microcycle chronic period, based on a small standardized ES (0.2 x SD) from the average number of minutes played in competition.

## Results

Two players were injured at the beginning of the 10 full competitive microcycles analyzed: the first athlete returned to full training in week 5 after meniscus surgery, and the second athlete returned to full training in week 6 after a groin injury. Two individuals suffered an injury within the 10 full competitive microcycles. The overall injury rate during the 10-microcycle seasonal period was 5.4/1,000 h including contact and non-contact injuries.

### Spikes in ACWR

**Figure 1:**
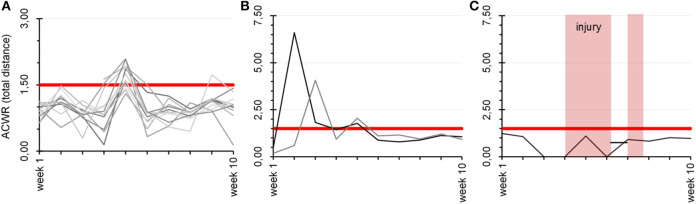
Visual representation of individual ACWR development over all monitored training weeks for TD. **(A)** Refers to all players who did not suffer a non-contact injury. **(B)** Represents two players who showed considerably higher ACWR in week 5 and 6, respectively, but did not suffer a non-contact injury (players 6 and 12). **(C)** Shows the ACWR development of the player who did suffer a non-contact injury in week 5. Red shaded areas highlight the time of training interruption for player 3. In all three figures the critical ACWR of 1.5 is marked by the red line.

Visual representation of individual ACWR courses over the training weeks is provided in Figure 1 for TD as an example. Athletes 6 and 12, after full integration in team training, demonstrated higher ACWRs in weeks 5 and 6, respectively, compared to the overall mean, but they did not suffer injuries.

Table 1 shows the averages and spikes in ACWR for players who did not suffer an injury but exceeded the critical threshold of an ACWR > 1.5 over the 10-microcycle chronic period. This is equivalent to 1 player exceeding the critical threshold for ACWR in TD, 2 players for ACWR at MSD, 2 players for ACWR at HSD, 2 players for ACWR at VHSD, and 2 players for ACWR in DC at sprint per week.

**Table 1:** Averages and spikes per player in ACWR over the 10-microcycle chronic seasonal period. Data are mean ± standard deviation.

	**TD**	**MSD**	**HSD**	**VHSD**	**Sprinting**
Player 1	1.04 ± 0.45 ^(1)^	1.04 ± 0.74 ^(1)^	1.03 ± 0.74 ^(2)^	1.00 ± 0.73 ^(2)^	1.05 ± 0.91 ^(3)^
Player 2	1.01 ± 0.26 ^(1)^	0.97 ± 0.30 ^(1)^	0.94 ± 0.30 ^(1)^	0.94 ± 0.50 ^(2)^	0.91 ± 0.47 ^(1)^
Player 3	1.05 ± 0.35 ^(1)^	1.07 ± 0.43 ^(2)^	1.08 ± 0.46 ^(2)^	1.06 ± 0.51 ^(1)^	1.02 ± 0.46 ^(1)^
Player 4	1.03 ± 0.39 ^(1)^	1.04 ± 0.70 ^(1)^	1.01 ± 0.65 ^(1)^	1.00 ± 0.73 ^(1)^	1.04 ± 0.85 ^(2)^
Player 5	1.04 ± 0.54 ^(2)^	1.08 ± 0.78 ^(2)^	1.06 ± 0.80 ^(2)^	1.07 ± 1.02 ^(2)^	1.21 ± 1.57 ^(2)^
Player 6	1.04 ± 0.31 ^(1)^	1.02 ± 0.39 ^(2)^	1.03 ± 0.44 ^(2)^	1.04 ± 0.62 ^(2)^	0.91 ± 0.54 ^(2)^
Player 7	1.00 ± 0.31	1.00 ± 0.40 ^(1)^	0.99 ± 0.41 ^(1)^	1.01 ± 0.47 ^(1)^	1.01 ± 0.88 ^(2)^
Player 8	1.02 ± 0.46 ^(1)^	1.01 ± 0.67 ^(2)^	0.97 ± 0.66 ^(2)^	0.92 ± 0.57 ^(2)^	0.87 ± 0.41
Player 9	1.02 ± 0.31 ^(1)^	1.03 ± 0.36 ^(1)^	0.99 ± 0.27	0.94 ± 0.25	0.91 ± 0.38 ^(1)^
Player 10	1.01 ± 0.24 ^(1)^	1.01 ± 0.28 ^(1)^	0.99 ± 0.28 ^(1)^	0.97 ± 0.40 ^(1)^	0.97 ± 0.50 ^(2)^
Player 11	1.05 ± 0.26 ^(1)^	1.03 ± 0.35 ^(2)^	1.01 ± 0.31	1.02 ± 0.36 ^(1)^	1.07 ± 0.50 ^(3)^
Player 12	1.30 ± 1.09 ^(2)^	1.38 ± 1.29 ^(2)^	1.35 ± 1.07 ^(3)^	1.23 ± 0.74 ^(3)^	1.10 ± 0.58 ^(2)^
Player 13	1.45 ± 1.91 ^(3)^	1.86 ± 2.76 ^(5)^	2.47 ± 3.15 ^(5)^	3.39 ± 6.15 ^(3)^	2.19 ± 2.59 ^(4)^

*TD, Total distance; DC, Distance covered; MSD, DC above moderate speed (>14.4 km/h); HSD, DC at high speed (>18.0 km/h); VHSD, DC at very high speed (>21.0 km/h); DC sprinting (>24 km/h); (1) to (5): number of spikes (danger zone) per player during the 10-microcycle chronic seasonal period without a subsequent injury*.

Only two individuals suffered an injury during the 10 full competitive microcycles. The player who did suffer a non-contact injury exceeded the critical threshold only once, by 0.6 for ACWR in DC at sprint 2 weeks before the injury. No further values > 1.5 were obtained for ACWR in the different running activity parameters for these athletes.

### ACWR and Injuries

Average ACWRs of players without injury over all weeks were 1.08 ± 0.47 for TD, 1.12 ± 0.67 for MSD, 1.15 ± 0.77 for HSD, 1.20 ± 1.56 for VHSD, and 1.10 ± 0.62 for DC at sprint. Two individuals suffered an injury during the 10 full competitive microcycles. Neither individual had exceeded the 1.5 ACWR threshold in the 4 weeks prior to the injury event:

The first athlete experienced a non-contact hamstring strain injury resulting in a 3-weeks training interruption. The average ACWR for this player was 1.07 ± 0.15 for TD, 1.06 ± 0.24 for MSD, 1.04 ± 0.31 for HSD, 0.99 ± 0.38 for VHSD, and 0.89 ± 0.46 for DC at sprint. Within the 4 weeks prior to his muscle strain injury, the ACWR ranged from 0.31 (at sprint) to 1.07 in TD.

The second athlete suffered a contact injury concussion; he missed 2 weeks of training. The average ACWR was 0.94 ± 0.46 for TD, 0.91 ± 0.33 for MSD, 0.92 ± 0.42 for HSD, 0.90 ± 0.51 for VHSD, and 0.74 ± 0.42 for DC at sprint. The ACWR for this player ranged from 0.41 (at sprint) to 1.41 (TD) 4 weeks prior to injury.

### Imbalances in Chronic Training Load

The training contents of each week were operationalized by multiple external load variables. Their coefficients of variation when averaged across all sessions were 29.0% for TD, 41.2% for MSD, 44.4% for HSD, 54.7% for VHSD, and 72.8% for DC at sprint.

**Figure 2:**
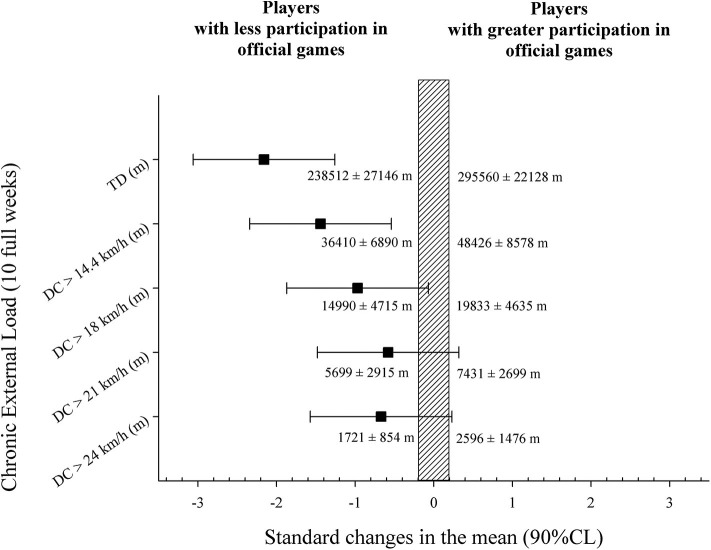
Differences in total external load during the 10-microcycle chronic period between players with lesser and greater participation in official games. Squares represent standard changes (mean) with 90% CL. Percent changes refer to the differences between competition and training sessions ± standard deviations. ES refers to the effect size.

Figure 2 shows the differences in total external load for training sessions and official matches during the 10-microcycle chronic period between players with lesser and greater participation (*small* difference*, p* < 0.05) in official games. Players with fewer minutes in competition covered a significantly lower TD (-19.6 ± 9.9%, very large ES) and DC > 14 km/h (−24.8 ± 19.5%, very large ES) over the course of the 10-week period in comparison with the players with greater participation in official games (*p* < 0.05). A reduction in the HSD (−25.1 ± 29.3%, moderate ES), VHSD (−25.5 ± 45.6%, moderate ES), and DC at sprint (−30.6 ± 64.9%, moderate ES) was also shown in the players with a significantly smaller number of minutes in competition.

## Discussion

This study offers a major insight into player monitoring and individual load variations within a competitive season in conjunction with the occurrence of contact and non-contact injuries. We found that (i) spikes in ACWR were not associated with injury incidence, and spike magnitude and distribution varied strongly between athletes, with ratios frequently exceeding 1.5; (ii) significant differences in external load between players with greater or lesser exposure to matches existed, causing an imbalance in the external load values during a 10-microcycle chronic period; and (iii) two athletes who experienced an injury showed no history of an augmented ACWR > 1.5 in any of the recorded parameters within the weeks prior to the injury.

Studies have examined the association between external/internal training load and injury in professional soccer players, with highly inconsistent findings ranging from significant interrelationships to clear disassociations (Hulin et al., 2016; Bowen et al., 2017, 2020; Malone et al., 2017; Fanchini et al., 2018; Lolli et al., 2019). Using the external load to describe the relationships between ACWR and injury risk, Hulin et al. (2016) found that the ratio of acute to chronic workloads (absolute total distance) predicted injuries in elite rugby players. Bowen et al. (2017), working with elite youth football players, showed that three-weekly accelerations were the strongest predictor of injury risk. The same authors in 2019, working with professional soccer players, found that a number of GPS-derived spikes in ACWR were associated with injury risk (except high-speed and sprint distance), while spikes in decelerations were associated with the greatest non-contact injury risk. In contrast, Malone et al. (2017) suggested that coaches must expose players to high percentages of maximal speed during training sessions as a potential “vaccine” against subsequent soft tissue injury. In addition to this, several limitations exist in professional soccer for the assessment of GPS-derived external loads during the whole season (Buchheit, 2017). Limitations such as integrating data from different tracking systems (GPS during training vs. semiautomatic cameras for games), international breaks without data from national teams or using systems that differ from those used in the club, or predicting data when nothing is available, make the application of this model very difficult in professional soccer (Buchheit, 2017). While session-RPE may offer some advantages for data collection compared to external load in the elite soccer context, recent articles using internal load indicators suggest a poor interrelationship between the ACWR and injury incidence (Fanchini et al., 2018; Lolli et al., 2019). The present results did not find any association between spikes in ACWR quantified from GPS with injury incidence during the 10-microcycle seasonal period. In the current study, 13 soccer players who did not suffer an injury repeatedly exceeded the critical threshold of an ACWR > 1.5 (i.e., an average of once per player for TD, or twice per player for DC at high speed). Previous studies showed that players with optimal levels of intermittent fitness are able to complete increased weekly high-speed distances with a reduced injury risk (Malone et al., 2018), stronger athletes were shown to tolerate spikes in workload better than weaker athletes, identifying faster speed, repeated-sprint ability and higher relative lower body strength as potential moderators of a subsequent injury (Malone et al., 2019). In the present study, players supplemented the soccer training with a strength-training program structured in different session types throughout the week during the entire competitive season. Most probably, this regular strength training has positive influenced, along with the optimal intermittent fitness, in tolerate the multiple spikes in workload during the 10-microcycle seasonal period. Conversely, the only muscle injury during the period was recorded in the fastest player after showing no signs of overload within the 4 weeks prior to his muscle strain injury. As practitioners, we also observed a succession of spikes in the ACWR after sickness or injuries in athletes reintegrating into regular training schedules. For those injured players, the previous weeks preceding the spikes were often devoted to rehab and return-to-play activities, which are typically associated with a reduced training volume and intensity. This will obviously lead to elevated ACWRs when returning to normal team training. Thus, a possible option for previously injured players could be to eliminate the return-to-play window from the general analysis. Nevertheless, our results showed that these players did not suffer an injury after very large spikes in training load.

In addition, the results of the current investigation showed that when the running speed is higher (sprinting) it causes an increase in the number of false positives. This is most probably because the GPS-derived parameters are less accurate with increasing speed of movement (Rampinini et al., 2015; Al Haddad et al., 2018) and high-speed locomotor performance may not be a good indicator of players' physical performance (Mendez-Villanueva and Buchheit, 2011); consequently, this parameter may also be insufficiently reliable to predict future injuries. Two athletes who experienced a contact and non-contact injury, respectively, exhibited no signs of overload, as indicated by a constant ACWR alternating around 1.0 in the 4 weeks prior to the accident. In addition, players 6 and 12, as a result of lower loads during the rehabilitation and return-to-play process, showed clearly higher ACWRs compared to the overall mean after full training integration, but both athletes who had recovered from their injuries did not suffer subsequent injuries. Therefore, based on our results, despite the very low number of subjects and injured athletes, high ACWRs in external load parameters were not associated with injuries in professional soccer players.

Variability of the external load during training sessions or matches [evidenced by the coefficient of variation [CV] of a measurement] is of great significance for scientists and practitioners in avoiding biased interpretation when assessing differences during chronic training loads (Al Haddad et al., 2018). The results of the present study demonstrated that the CV for GPS-derived week-to-week variability of external load data ranged from 29 to 73%, depending on the parameter. A recent study (Martin-Garcia et al., 2018) with professional soccer players showed that the CV for weekly external load parameters averaged across all training sessions ranged from 20% for TD to >85% for distances covered at high speeds or sprinting. Likewise, (Al Haddad et al., 2018) revealed that GPS-derived match-to-match variability during official games' external load was particularly high for high-speed running or variables such as accelerations and decelerations. These variances are most probably caused by the complexity of movement patterns (Rampinini et al., 2015; Al Haddad et al., 2018) and factors associated with the reliability of the devices (Paul et al., 2015; Andrzejewski et al., 2016). For instance, a few steps at high acceleration can barely be tracked by GPS with the required precision (Paul et al., 2015), but these concentric–eccentric movements do account for the majority of non-contact muscle strain injuries (Schache et al., 2009).

### Imbalances in Chronic Loads

The results of the present study showed that participation in official games caused imbalances in the chronic external loads between players in a squad, with a significant reduction in the external chronic load of players with fewer minutes in competition (this ranged from −19 to −31%, depending on the running parameters). Accordingly, Stevens et al. (2017) reflected that nonstarter training sessions showed, in general, a lower load than regular training, with a considerably lower total weekly load in comparison to starters, especially in terms of high-speed running (Stevens et al., 2017). A recent study (Paulauskas et al., 2019) referred to the same phenomenon and identified weeks that could predispose players to unwanted spikes and adjusted player workload according to playing time. When there are imbalances in workload and players with less participation in games return to the starter team, the acute load will probably be substantially higher than chronic loads, predisposing the players to spikes in ACWR. In order to minimize these imbalances in workloads, the locomotor training stimuli during the compensatory training sessions should be close to reproducing the individual demands during competition in players with less exposure to the most important and specific stimuli induced by competitive games. Accordingly, the challenge for practitioners is to construct adequate soccer drills closer to competition. To minimize imbalances in chronic external loads within a squad, an interesting option is to use friendly or conditioned matches instead of regular training (Eniseler, 2005). Usually this may not to be possible due to the reduced number of players during this type of training session, but occasionally it could be possible to use players from the reserve team or organize a match against an external opponent.

### Limitations

Given the reality of professional soccer, only 14 full consecutive microcycles could be monitored with GPS during training sessions and official games throughout the whole in-season period (mainly due to international breaks, among other factors). Relative risk was not calculated to examine the relationships between injury risk and spikes in workload because there were not enough accumulated injuries during the 10-microcycle seasonal period. Another limitation was the sample size, because some players trained with the first team on some occasions and were excluded from the analysis (which was thus missing some data).

## Conclusion

Injury prevention in elite team sports cannot be limited to the monitoring of a single ACWR number but should rather consider a variety of parameters associated with physical overload or residual exhaustion, in addition to signs of psychological fatigue (Buchheit, 2017). The present study demonstrated that spikes in the ACWR were not related to a subsequent injury occurrence in professional soccer players and a more holistic approach to fitness, fatigue and workload surveillance in athletes would be worthwhile (Bahr, 2016). Substantial differences in participation in official games caused significant imbalances in the chronic external loads between players in a squad, which should be minimized in training sessions in order to prevent substantial changes in workload for those who usually do not play (i.e., substitutes, and players not on the match list).

## Data Availability Statement

All datasets generated for this study are included in the article/supplementary material.

## Ethics Statement

Ethical review and approval were not required for the study on human participants in accordance with the local legislation and institutional requirements. Written informed consent to participate in this study was provided by the participants.

## Author Contributions

Individual contributions to the paper using the relevant CRediT roles. LS-A, MR, and RR: conceptualization. BD, SS, and KF: data curation. LS-A, BD, MR, IT, SS, KF, and RR: formal analysis, resources, and roles/writing—original draft. LS-A, BD, and IT: investigation and software. LS-A, BD, MR, SS, and RR: methodology. RR: supervision. MR, IT, and SS: validation. LS-A and MR: visualization. LS-A, MR, KF, and RR: writing—review and editing. n.a: funding acquisition. All authors contributed to the article and approved the submitted version.

## Conflict of Interest

LS-A and IT were employed by company FC Basel 1893. BD was employed by company FC Sevilla. The remaining authors declare that the research was conducted in the absence of any commercial or financial relationships that could be construed as a potential conflict of interest.
